# Implementation of methadone therapy for opioid use disorder in Russia – a modeled cost-effectiveness analysis

**DOI:** 10.1186/s13011-016-0087-9

**Published:** 2017-01-20

**Authors:** Bulat Idrisov, Sean M. Murphy, Tyler Morrill, Mayada Saadoun, Karsten Lunze, Donald Shepard

**Affiliations:** 1 Department of Medicine, Section of General Internal Medicine, Boston Medical Center, 801 Massachusetts Avenue, 2nd Floor, Boston, MA 02118 USA; 2 Department of Health Policy and Administration, Washington State University, Spokane, WA 99202 USA; 3 Schneider Institutes for Health Policy Heller School, Brandeis University, Waltham, MA 02454 USA

**Keywords:** HIV treatment, Russia HIV, Drug dependence, TB, Methadone

## Abstract

**Background:**

Opioid agonist therapy using methadone, an effective treatment of opioid use disorders (OUD) for people who inject drugs (PWID), is recommended by the World Health Organization as essential to curtail the growing HIV epidemic. Yet, despite increasing prevalence of OUD and HIV, methadone therapy has not yet been implemented in Russia. The aim of this modeling study was to estimate the cost-effectiveness of methadone therapy for Russian adults with a diagnosed OUD.

**Methods/Design:**

We modeled the projected program implementation costs and estimated disability-adjusted life years (DALYs) averted over a 10-year period, associated with the provision of methadone therapy for a hypothetical, unreplenished cohort of Russian adults with an OUD (*n* = 249,000), in comparison to the current therapies at existing addiction treatment facilities. Our model compared four distinct scenarios of treatment coverage in the cohort ranging from 3.1 to 55%.

**Results:**

Providing methadone therapy to as few as 3.1% of adults with an OUD amounted to an estimated almost 50,000 DALYs averted over 10 years at a cost of just over USD 17 million. Further expanding service coverage to 55% resulted in an estimated almost 900,000 DALYs averted, at a cost of about USD 308 million.

**Conclusion:**

Our study indicated that implementing opioid agonist therapy with methadone to treat OUD at existing facilities in Russia is highly cost-effective.

## Background

After the fall of the Soviet Union and formation of the Russian Federation, smuggling routes of opium were established from Afghanistan through Russia [18]. These routes increased the availability of opioid-based drugs in Russia, mostly heroin, leading to rapid increases in the number of people who inject drugs (PWID) and thus the spread of HIV infection. Presently, 1.8% of Russians inject drugs [30, 45].

With rapid increase in injection drug use in Russia, the number of new HIV infections grew 5.3% on average in the past 10 years, making it one of the highest HIV infection rates in the world [39, 45, 47]. Moreover, substance use, HIV, and tuberculosis frequently co-occur in the country [23]. The HIV epidemic in Russia is primarily driven by injection drug use; up to 70% of HIV positive Russians have been infected through injection drug use [1, 9]. This is seven times the world average of 10% [29]. Based on the latest report from the Russian Ministry of Health, 540,000 PWID currently live in Russia, and 40,500 are hospitalized annually for opioid use disorder (OUD) based on International Statistical Classification of Diseases and Related Health Problems (ICD) 10 standards [20]. In 2015, a mere 14% of HIV-positive Russians received antiretroviral therapy (ART), placing the country among the lowest in the world with regard to ART rates among those infected with HIV [45]. Among PWID, access to HIV care is even disproportionately lower, but improving narcology services is an opportunity, since appropriate addiction care has shown to improve engagement in HIV treatment in other parts of the world [40, 47].

One of the major factors contributing to the growing HIV epidemic in Russia is the lack of evidence-based prevention and treatment programs for both HIV and substance use directed to populations at risk, such as PWID. Because opioid agonist therapy (OAT) using methadone or buprenorphine is not legal in Russia and funding for effective, legal programs such as needle and syringe exchanges is limited, it is assumed that many preventable HIV infections occur each year in Russia [26, 27, 32].

The standard of care at Russian inpatient narcology hospitals consists of diagnostic procedures, detoxification, and rehabilitation. Detoxification typically occurs within the first week of hospitalization. The process, which occurs mostly at inpatient setting, typically utilizes the following medications for opioid withdrawal therapy: antidepressants, antipsychotics, anticonvulsants, non-opiate analgesics, adrenergic, and alfa 2 agonist. The only available medication in Russia that has been shown to be effective at preventing opioid use relapse is the opiate antagonist naltrexone, which is generally only administrated upon discharge from inpatient care [21, 22, 32]. During inpatient hospitalization, patients receive drug counselling and treatment for comorbid psychiatric conditions, but integration to other treatment modalities such as HIV care is limited [14, 19]. PWID are encouraged to receive monthly outpatient care where they could receive psychotherapy and referral for treatment of comorbid conditions. However, linking and retaining patients in care is challenging, as narcology hospitals are limited in their capability to provide evidence-based pharmacological treatment, and their patients are subject to immense stigmatization [26].

OAT using methadone or the partial agonist buprenorphine is regarded to be the most effective treatment for opioid use disorders and beneficial as a harm reduction program to reduce HIV risks [25, 31]. The World Health Organization (WHO) has endorsed OAT and harm reduction. Methadone, included in its list of essential medications, was already used by 59 countries worldwide in 2009 [48]. In addition to being an effective therapy for OUD, methadone has also been shown to provide advantageous economic value [34]. PWID are administered methadone treatment to reduce or eliminate cravings and withdrawal symptoms of illicit opiates. Methadone acts as a full agonist of mu (μ) receptors in neurons acting via inhibitory G protein resulting in presynaptic inhibition of neurotransmitters [46]. Methadone is a full agonist and 3 times more potent than morphine with a 1–3 day half-life, which makes it suitable for use in harm reduction programs (see graph 1 for list of desirable indirect effects of OAT).

Despite the multiple benefits associated with OAT, it has not yet been accepted in the Russian health system. Determining the potential benefits of an OAT program in Russia is relevant, as opioid use disorders and HIV infections associated with drug use are likely to continue to increase until effective OUD treatment programs are introduced [3, 5].

The aim of this paper is to estimate the cost-effectiveness attributable to the hypothetical introduction of methadone therapy for Russian adults with a diagnosed opioid use disorder.

## Methods

### Overview

We modeled the cost and disability adjusted life years (DALYs) averted associated with the provision of methadone therapy for a hypothetical, unreplenished cohort of Russian adults with an OUD, relative to the current system where OAT is legally banned. According to a 2016 Russian Ministry of Health report, the number of opioid users who met OUD criteria was approximately 249,000 nationwide, most of whom are injection drug users [20]. As recommended in the World Health Organization’s Guide to Cost-Effectiveness Analysis [10], cost (converted to 2015 U.S. Dollars) and outcomes (DALYs) were modeled over a 10-year horizon. Cost and DALYs occurring beyond 1 year were discounted at the recommended yearly rate of 3% to account for time preference [10].

Although partial agonists such as buprenorphine are also used effectively to treat opioid dependence worldwide, our analyses focus on methadone only; however, we believe that all options should be explored for patients. Methadone therapy (MT) has typically been shown to be the most economically viable option, with rates of effectiveness that are at least as good as those for buprenorphine maintenance therapy, at a lower cost [35]. Thus, methadone therapy would be a logical choice for implementing a large-scale OAT program. We interpreted our results in the context of the cost-effectiveness thresholds recommended by the World Health Organization’s Choosing Interventions that are Cost–Effective project (WHO-CHOICE). They state that interventions capable of averting one DALY for < 3x the per-capita gross domestic product (GDP) should be considered “cost-effective”, while those capable of doing so at a cost below the per-capita GDP should be considered “highly cost-effective” [49].

### Methadone therapy for OUD: initiation and retention

We considered several scenarios for implementing a national methadone therapy program. Similar to Alistar et al. [2], we used a “low” scenario where treatment would be available for 3.1%, a “moderate” scenario with slots for 12.5%, and a “high” scenario with slots available for 25%. We also considered a scenario with slots available for 55% of the adult OUD population, which was based on the maximum coverage in Vietnam following the government’s decision to expand OAT programs upon successful completion of their pilot program [36]. Methadone therapy retention rates were also based off findings from the first Vietnamese MT cohort, where the response rate was 89.9% after 9 months [42].

### Effectiveness measurement

Effectiveness of the proposed rate of methadone therapy coverage was measured by calculating DALYs averted. The DALY is a global measure of disease burden with possible scores ranging from 0 (no disability/ impact) to 1 (one year of “entirely healthy life” lost). Similar to the quality-adjusted life-year (QALY), the DALY is a generalizable measure of effectiveness, which permits comparisons across diseases and interventions. DALYs are calculated by adding Years of Life Lost (YLL) and Years Lost due to Disability (YLD) [10]. YLL were calculated by multiplying the number of deaths in a year by the average number of years of life lost due to opioid use. For YLL, we used the all-cause mortality rates estimated by Chang et al. [7], both for patients who used heroin and were in opioid substitution therapy, 15.5 per 1000 person years, and those who were not in opioid substitution therapy, 23.9 per 1000 person years. We then multiplied these figures by the respective average number of years of life lost, which were 10.6 for patients who used heroin and were on methadone therapy and 18.6 for those who were not [7]. To calculate YLD, we first estimated the prevalent number of individuals with an OUD and a comorbid HIV infection, and the number with an OUD and comorbid HIV and tuberculosis (TB) infections, as well as the number with an OUD who did not have these comorbidities. Prevalence of co-occurring OUD and HIV was obtained from a systematic review performed by Jolley and colleagues [17], who found that approximately 30% of Russian PWID have HIV. The number of individuals with comorbid OUD, HIV, and TB was estimated by averaging prevalence rates from three studies which found TB rates of 17%, [13] 18.2% [11] and 20.3% [33] among HIV infected patients, which yielded a final estimate of 18.5%. The estimated number of prevalent cases for each disorder/disease state was then multiplied by the associated disability weight obtained from Salomon et al. [38], with disability weights for comorbid states calculated using the multiplicative approach [15].

### Cost measures

The per-person treatment costs were taken from Tran et al. [43], who estimated the average cost of running Vietnam’s methadone therapy program from the perspective of their national healthcare system to be $278 [2015 USD]. We assumed costs in the Russian Federation, likewise an (upper) middle-income country, to be similar, if not lower once implemented.

## Results

Table 1 displays the estimated present-value costs and DALYs averted for each of the four scenarios. The “low” scenario of supplying OAT to 3.1% of adults with an OUD resulted in a present value of 49,739 DALYs averted at a total cost of $17,068,524 over the modeled 10-year horizon. Expanding the number of opioid substitution slots to 12.5% of the OUD population resulted in present value estimates of 201,234 DALYS averted at a cost of $69,051,186 over 10 years. The scenarios where slots are available to 25% or 55% of the OUD population resulted in present value estimates of 404,265 and 898,958 DALYs averted, respectively, at costs of $138,707,623 and $308,382,234, respectively.

**Table 1:** Predicted costs and effectiveness outcomes

	MT supplied to 3.1% of OUD population		MT supplied to 12.5% of OUD population		MT supplied to 25% of OUD population		MT supplied to 55% of OUD population		
Year	DALYs^a^Averted	Cost^a,b^	DALYs^a^Averted	Cost^a,b^	DALYs^a^Averted	Cost^a,b^	DALYs^a^Averted	Cost^a,b^	Cost/DALY Averted^a,b^
1	6,562	2,145,882	26,458	8,652,750	52,916	17,305,500	116,415	38,072,100	327
2	6,148	2,034,130	24,810	8,208,772	49,674	16,435,185	109,568	36,250,553	331
3	5,759	1,928,198	23,262	7,787,574	46,626	15,608,638	103,115	34,516,158	335
4	5,395	1,827,783	21,808	7,387,988	43,759	14,823,660	97,034	32,864,744	339
5	5,053	1,732,597	20,441	7,008,905	41,064	14,078,160	91,304	31,292,342	343
6	4,731	1,642,368	19,158	6,649,274	38,531	13,370,151	85,905	29,795,170	347
7	4,430	1,556,838	17,953	6,308,095	36,149	12,697,749	80,819	28,369,630	351
8	4,147	1,475,762	16,822	5,984,422	33,910	12,059,164	76,027	27,012,295	356
9	3,882	1,398,908	15,759	5,677,358	31,806	11,452,693	71,512	25,719,901	360
10	3,633	1,326,057	14,762	5,386,049	29,829	10,876,723	67,260	24,489,341	365
Present value	49,739	17,068,524	201,234	69,051,186	404,265	138,707,623	898,958	308,382,234	343

^a^Discounted at 3% after year 1; ^b^2015 US Dollars

## Discussion

Unfortunately, the care for patients with an OUD in Russian narcology hospitals is associated with low successful treatment outcomes and follow up rates [24, 32]. To our knowledge, this study is the first to estimate the effectiveness of implementing an evidence-based form of OAT in Russia, as well as the associated costs. Results indicate that a national methadone program could be implemented in Russia at a cost-per-DALY-averted of $343 [2015 USD]. Given that Russia’s 2015 per-capita GDP was $9057 [41], these estimates are well below the cost-effectiveness thresholds recommended by the WHO-CHOICE framework, indicating that implementing an OAT program in Russia would be “highly cost-effective”.

Although OAT is not yet available in Russia, in our opinion the infrastructure partially exists to implement such programs [50, 51] and unlike most countries facing a similar challenge of HIV and substance use epidemics, Russia has a sufficient number of qualified health care cadres and institutions [37]. Free healthcare in Russia is a constitutional right; however, patients may only use their mandatory insurance coverage at public hospitals since private-based hospitals rarely contract with the Mandatory Health Insurance Fund (MHIF). Russian citizens contribute to MHIF by way of mandatory payments though general taxation and payroll contributions. The model of service delivery in Russia is built as a multi-level system with different networks of healthcare facilities organized by specialty (e.g. primary care, HIV, TB, addiction) and geographical location [37]. The levels consist of rural/small-district outpatient facilities, central rural/municipal hospitals, regional hospitals and federal hospitals [8, 37]. Substance use disorder is treated at specialized narcology hospitals that also consist of district level outpatient facilities and larger inpatient narcology hospitals. Given that these facilities are already built and functioning, start-up costs for an OAT program would likely be minimal. Modalities of provision could be derived from former Soviet countries with similar health system infrastructures, such as Ukraine, where OAT exists and has been demonstrated to improve care quality and quality of life among HIV infected PWID, especially if integrated with HIV care [4, 28].

There are several limitations to our study. First, we used a prevalence-based model, as opposed to a state-based model, which allows individuals to transition from one health state to another over the modeled time period, according to estimated transition probabilities. Furthermore, we were unable to estimate the number of individuals with HIV who were on ART, which limits our ability to incorporate the benefits of increased use of ART associated with adherence to OAT [44]. When calculating DALYs, we used a conservative approach and calculated YLDs using the disability weight of 0.221 associated with “HIV: symptomatic, pre-AIDS” versus the 0.547 weight associated with “AIDS: not receiving ART”. Increased ART adherence has a positive impact on the number of DALYs averted [6, 12]. This is especially relevant in locations with high rates of HIV among people who use drugs and low ART coverage, such as in St. Petersburg where 43% of PWID are HIV positive with low treatment continuum [16]. Future studies on effectiveness of an OAT program in Russia should also focus on data validity. For example, many of the prevalence rates built into the model, as well as the estimated cost of implementing the methadone program, were derived from studies performed in other countries due to a lack of such information in Russia. Finally, like any model, our study does not entirely reflect all costs and benefits to society that may be associated with the implementation of an OAT program. That is, prior studies have shown evidence of cost-offsets associated with effective OAT, resulting from reduced use of high-cost healthcare services (e.g., the emergency department) and non-health related outcomes such as reduced criminal activity [35].

## Conclusion

This study suggests that implementing a methadone therapy program in Russia would be highly cost-effective due to its effectiveness in averting DALYs among people who inject drugs. Russia’s robust health workforce and its constitutionally assured right to health provide a formidable implementation framework for updating the Russian addiction treatment and rehabilitation standards to include international, evidence-based addiction treatment with opioid agonist medication. This will reduce the burden of disease related to substance use and associated conditions such as HIV and TB. By reconsidering its position on opioid agonist therapy, Russia could create substantial progress in the country’s fight against the burden of, OUD, HIV and other drug-related diseases, as well as the human tragedies behind them.

## Abbreviations

ART: Antiretroviral therapy
DALY: Disability adjusted life years
GDP: Gross domestic product
ICD: International Statistical Classification of Diseases and Related Health Problems
MHIF: Mandatory Health Insurance Fund
MT: Methadone therapy
OAT: Opioid agonist therapy
OUD: Opioid use disorder
PWID: People who inject drugs
YLD: Years lost due to disability
YLL: Years of life lost
